# A Sustainability-Based Expert System for Additive Manufacturing and CNC Machining

**DOI:** 10.3390/s23187770

**Published:** 2023-09-09

**Authors:** Josage Chathura Perera, Bhaskaran Gopalakrishnan, Prakash Singh Bisht, Subodh Chaudhari, Senthil Sundaramoorthy

**Affiliations:** 1 Industrial and Management Systems Engineering, West Virginia University, Morgantown, WV 26506, USA; chathurajp93@icloud.com (J.C.P.); psb00008@mix.wvu.edu (P.S.B.); 2 Oak Ridge National Laboratory (ORNL), 1 Bethel Valley Rd, Oak Ridge, TN 37830, USA; chaudharisa@ornl.gov (S.C.); sundaramoors@ornl.gov (S.S.)

**Keywords:** sustainability, additive manufacturing, CNC machining, binder jetting, direct metal laser sintering, expert system

## Abstract

The objective of this research study is to develop a set of expert systems that can aid metal manufacturing facilities in selecting binder jetting, direct metal laser sintering, or CNC machining based on viable products, processes, system parameters, and inherent sustainability aspects. For the purposes of this study, cost-effectiveness, energy, and auxiliary material usage efficiency were considered the key indicators of manufacturing process sustainability. The expert systems were developed using the knowledge automation software Exsys Corvid^®^V6.1.3. The programs were verified by analyzing and comparing the sustainability impacts of binder jetting and CNC machining during the fabrication of a stainless steel 316L component. According to the results of this study, binder jetting is deemed to be characterized by more favorable indicators of sustainability in comparison to CNC machining, considering the fabrication of components feasible for each technology.

## 1. Introduction

Industrial manufacturing accounts for roughly 28% of energy use and 18% of greenhouse gas emissions in the United States [1]. Most modern manufacturing technologies operate with the aid of electrical power. In the United States, 60% of the electricity is generated from burning fossil fuels [1], which is widely known to negatively affect the environment. With the current trends in global warming and climate change, it is evident that immediate action is required to reduce the environmental impact caused by manufacturing. The integration of computer-aided manufacturing with machine tools has paved the way toward the birth of efficient technologies such as CNC (computer numerical control) machining and 3D printing/additive manufacturing (AM), which are predominantly used in today’s industrial sector. The introduction of these innovative technologies to production systems has redefined the manufacturing landscape in a way that has compelled users to investigate their sustainability. The most widely used description portrays sustainability as an intersection of the economy, society, and the environment [2]. Numerous studies have attempted to analyze and compare the sustainability aspects of AM and CNC machining through product life cycles but lack the consideration of equally feasible product, process, and system parameters for each technology. The existing decision support systems do not provide a simple framework that could be used to analyze and compare energy, cost, and auxiliary material use of AM and CNC machining.

**Figure 1:**
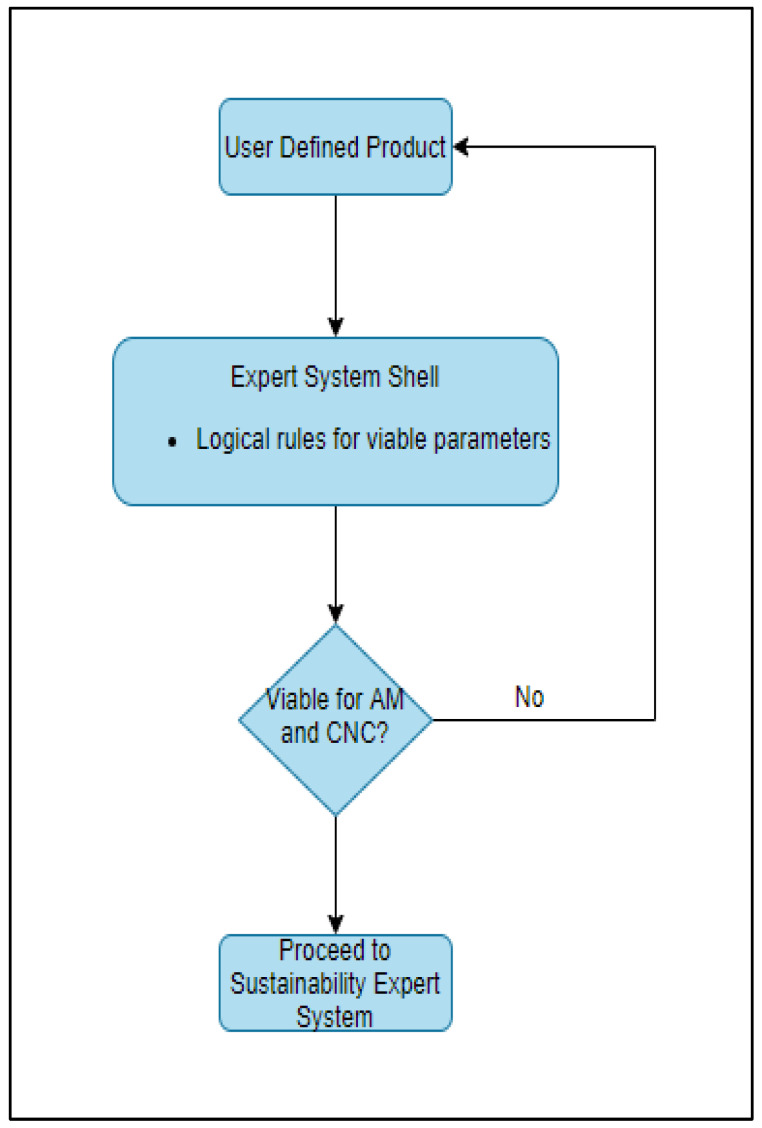
First expert system design.

**Figure 2:**
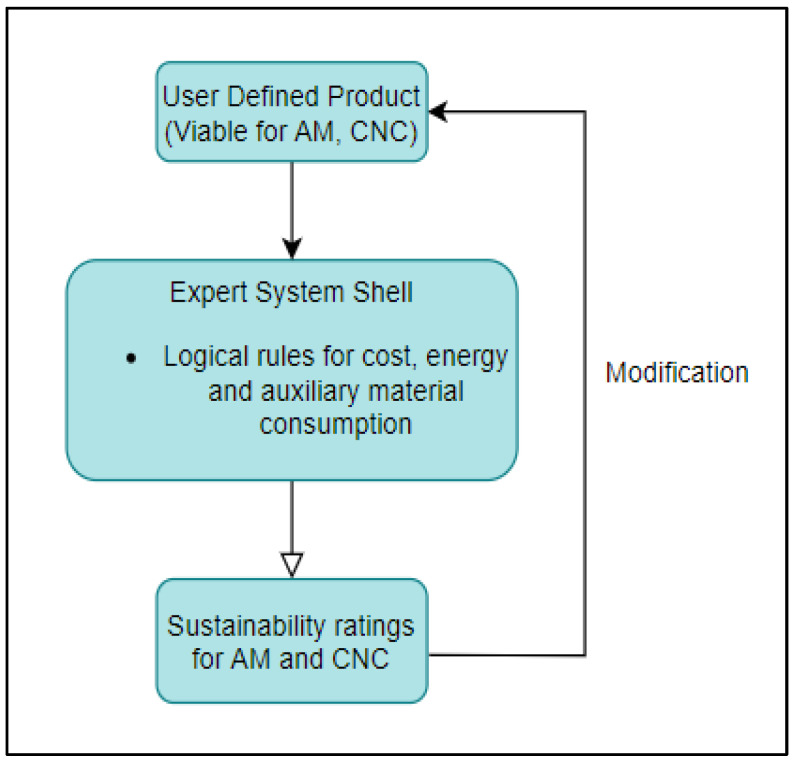
Second expert system design.

An expert system (ES) is a computerized artificial intelligence system, which replicates the decision-making ability of a human expert [3]. Expert systems comprise a knowledge base and an inference engine, and the resulting conclusions are the output of the expert system to information and criteria supplied by the user. This research study aims to develop a set of expert systems that can be used in the product development phase to gain insight into the feasibility/sustainability impact of metal AM and CNC machining. The research study was restricted to two types of AM (DMLS–direct metal laser sintering, and BJP–binder jetting process) and CNC machining, as each technology is capable of fabricating parts with comparable product parameters. A simplified depiction of an expert system design is shown in Figure 1 and Figure 2.

As shown in the figures above, the first expert system is used to evaluate if the user-defined component is within the feasible product, process, and system parameters of both CNC machining and AM. Once the primary comparative analysis is completed, the second expert system can be used to seek quantitative and qualitative advice on the sustainability impacts of the two processes. An optimal means of manufacturing, alongside environmental (energy, auxiliary material use, etc.) and financial metrics, is provided as the final output of the tool. With the rapid growth in generative artificial intelligence, the proposed methodology is an alternative option that can be further explored to aid sustainability advisory services.

## 2. Literature Review

A study titled “The Impact and Application of 3D Printing Technology” [4] discusses the current applications of 3D printing and the ways in which it has transformed manufacturing processes in various industries. Based on this paper, 3D printing has provided a way to decrease costs and lead time in most manufacturing setups while delivering products with higher quality and durability. A decision support model based on the analytic hierarchy process (AHP) was developed [5], which could be used to evaluate which AM technology would be most effective for manufacturing a particular part. The environmental impacts of additive manufacturing vs. traditional machining via life cycle assessment were compared in a previous study [6], which indicates that the primary difference between AM and CNC machining is that the latter typically uses cutting oil for lubrication, an additional source of waste.

A life cycle assessment and analysis of energy consumption was conducted in PBF (powder bed fusion) and DED (directed energy deposition) during the manufacturing of metal parts [7]. The authors further examined energy consumption reduction strategies such as layer thickness optimization and build volume maximization. In a case study evaluation of CNC and AM, the authors found that material and energy consumption throughout the life cycle are lower for AM.

An exploratory study showed the advantages, challenges, and implications of additive manufacturing on sustainability. As stated in [8], additive manufacturing has the potential to provide numerous sustainability advantages. These include the capability to optimize geometries and create lightweight components that reduce material and energy consumption, and inventory waste reduction due to the ability to create spare parts on demand. The impacts of process planning on the sustainability of CNC machining based on energy consumption, relative delay time, and machining costs were evaluated [9]. A case study was conducted to verify the developed strategies, which resulted in a 25% reduction in energy consumption due to the use of optimal process planning.

From the existing research, it is quite clear that the utilization of 3D printing in production systems has the potential to reduce carbon emissions and overall environmental impact. However, most research studies do not confine the comparison criteria to product and system parameters equally viable for each type of manufacturing technology. A comprehensive evaluation of the sustainability impacts of CNC machining and AM would further expand the AM knowledge base and assist manufacturers in selecting the most beneficial technology in terms of sustainability.

## 3. Research Approach

For this research work, the expert system was split into two segments to diversify the user base and minimize the computing power required to execute the program. The objective of the first expert system is to aid the user in ascertaining product, process, and system parameters viable for the technologies used for comparative analysis. Upon selecting a suitable product, the second expert system would portray the sustainability performance of each manufacturing process in terms of energy, cost, and auxiliary material usage.

### 3.1. Selection of Sustainability Indicators

Cost-effectiveness is a major consideration in the selection of sustainable manufacturing processes. Economic aspects pertaining to labor, material, electricity, and equipment were analyzed to provide users with a realistic depiction of the overall costs incurred during the fabrication processes of BJP, DMLS, and CNC machining. Energy consumption is another vital indicator, where the most energy-intensive aspects considered in CNC machining and AM are material processing and primary/secondary/post-processing stages of manufacturing. For the purposes of this research, the auxiliary material usage of BJP, DMLS, and CNC machining was also considered a key indicator of sustainability.

### 3.2. Evaluation of Feasible Product and System Parameters

To prevent partial results, the proposed expert systems were designed such that the comparative analysis would be carried out within the range of product and system parameters equally suited for both manufacturing technologies. Product parameters such as material properties, geometric complexity, dimensions, surface finish, and tolerance, as well as system parameters such as cycle time, and production quantity justifiable by unit cost, were considered. Cycle time was assumed to represent the production capacity of each manufacturing technology.

### 3.3. Creation of Knowledge Base

Information was collected by means of literature reviews and interactions with manufacturing experts to create a database of knowledge related to the feasibility and sustainability of each technology. Cost-based calculations were performed for manufacturing processes including labor, material, electricity, and equipment. The gathered information in the knowledge base was converted to logical rules by means of an expert system shell, such that appropriate inference mechanisms could be utilized to evaluate user input data related to production systems.

### 3.4. Weightage and Scoring Methodology for Auxiliary Material

AHP was utilized [10] to develop a rating system that depicts the sustainability of each manufacturing process in terms of auxiliary material use. AHP is a method used to solve complex decision problems in which multiple criteria are evaluated for the selection of an alternative. The initial step of AHP is to decompose the optimization problem into a hierarchy of criteria and alternatives, which can be individually inspected with respect to the goal. The algorithm systematically analyzes each alternative against the criteria using pairwise comparisons.

The pairwise comparisons are represented in the form of n x n reciprocal matrices, where n is the number of criteria considered. AHP has the capability of transforming observational/experimental data into numerical values. By determining the weight factors for each criterion/subcriterion, all elements within the hierarchy can be assessed alongside the alternatives. A rating system can be created using the calculated weights to depict the suitability of each alternative toward fulfilling the end goal.

The pairwise comparison matrices will be of the following form:

$$A=\left[\begin{array}{cccc} 1 & a_{12} & \ldots & a_{1n}\\ a_{21} & 1 & \ldots & a_{2n}\\ \ldots & a_{ji}=1/a_{ij} & 1 & \ldots \\ a_{n1} & \ldots & \ldots & 1 \end{array}\right]$$

The sustainability expert system logical rules were designed such that all quantitative and qualitative data pertaining to auxiliary material within the knowledge base were converted to the rating scale. The AHP algorithm is able to choose between AM and CNC machining with respect to the auxiliary material consumption for a user-defined product.

### 3.5. Expert System Shell

Exsys Corvid^®^, the knowledge automation software used in this research, facilitates the development of complex decision support systems, which can be executed on a web browser. The Exsys Corvid^®^ program provides an object-oriented structure that also incorporates a simple logical rule-building procedure (in terms of IF, AND, OR, and THEN statements), which can emulate the thinking process of a human expert. The program allows for the incorporation of a vast range of variable types such as dynamic lists, statics lists, numeric values, strings, dates, collections, and confidence levels. The logic-building platform enables the organization of blocks (consisting of rules and trees with related functions), which can be executed in a user-defined order. The command block builder allows the developer to select the method of inferencing (backward or forward chaining) utilized by the Corvid^®^ inference engine to derive the necessary variables that serve as final outputs of the system.

## 4. System Design

### 4.1. Knowledge Base Development

As per the requirements of this research study, the knowledge base was generated by obtaining qualitative/quantitative data through literature reviews and expert opinions. The developed database consists of knowledge related to the feasibility regions of product/process/system parameters, energy consumption during manufacturing and material processing, costs resulting from fabrication, and the impact of auxiliary material consumption on the sustainability of metal 3D printing and CNC machining.

#### 4.1.1. Viable Product, Process, and System Parameters

It was considered essential to identify the product parameters (material, geometric complexity, surface quality, hardness, strength, and dimensional accuracy) and system parameters (production quantity considering overall unit cost and cycle time based on process parameters) equally viable for each considered technology. Through extensive literature reviews, it was found that the material types valid for the comparison of these technologies are variants of stainless steel (in the form of solid metal powder and billets).

Since geometric complexity has a negligible impact on the resources required for additive manufacturing, the complexity factor is only applicable to products manufactured using CNC machining. As described by Valentan [11], higher volume-to-facet ratios depict lower geometric complexity, whereas lower ratios illustrate higher complexity. Based on the research of Wedlund et al. [12] and the analysis of the breakeven point in terms of geometric complexity, the volume-to-facet ratios and the corresponding complexity factors for CNC machining were modified as represented in Table 1, for the purposes of this research study. The assignment of complexity factors to the volume–facet ratio was based on the estimation of machine time difference due to each complexity level.

**Figure 3:**
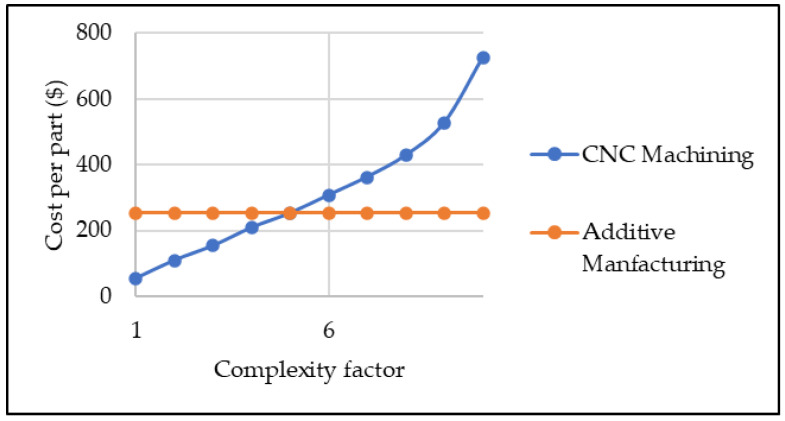
Average breakeven point for geometric complexity factor [12].

It is important to understand that the exact geometric complexity of products viable for both CNC machining and additive manufacturing is challenging to ascertain. However, by using subjective knowledge arising from existing research such as the studies of Varotsis [13,14] and the research study of Wedlund et al. [12], the equally viable geometric complexity factor (considering the overall cost per unit) was approximated to attain the value of 5, as shown in Figure 3.

The feasible material types were selected based on existing machine capabilities and specifications for BJP, DMLS, and CNC machining. With most stainless steel materials, CNC machining is capable of fabricating components with higher performance (in terms of quality and mechanical properties).

In production setups where either BJP has to be compared to CNC machining or DMLS to CNC machining, the customer expectations of component quality and mechanical properties are important considerations in selecting the most optimal technology. Therefore, the ranges for surface quality, hardness, strength, and tolerance describe the maximum possible component performance levels, which allow for the impartial comparison of BJP, DMLS, and CNC machining. If the required mechanical properties and quality of a component exceed the competency of current additive manufacturing technology, the manufacturing facility should opt for CNC machining.

According to the information provided by Varotsis [15], CNC machining and BJP are comparable when production quantities of less than 500 parts (of medium complexity) are required, and CNC machining and DMLS are comparable when the customer requirement is for less than 100 parts (of medium complexity.) In scenarios where the production quantity demanded by the customer exceeds the above-mentioned limits, manufacturing technologies such as injection molding should be considered.

In addition to considering production quantities justified by unit cost, it is crucial to verify if the selected process parameters of each technology can achieve the required cycle time. For CNC machining, BJP, and DMLS to be equally viable on a system level, the cycle times of the processes need to be equal to or less than the required cycle time as per the customer demands. The required cycle time (*T_c_*_(*required*)_) is calculated as follows:

$$T_{c\left(required\right)}=\frac{t_{required}}{P}$$

where

*t_required_* = the processing time to meet the required lead time (as per customer demand);
*P* = the production quantity demanded by the customer.

The processing time calculations for each manufacturing method were adopted from the work of Wedlund et al. [12] and Meteyer et al. [16]. However, the CNC machining time calculation was modified in order to account for the impact of the cutting speed, feed rate, and depth of the cut required for each operation (milling, drilling, and turning) on cycle time. For the purposes of this research, machining time was considered a suitable indicator of CNC toolpath for parts with varying geometric features. As an example, the time taken for milling can be calculated as follows:

$$t_{CNC\left(milling\right)}=\frac{V_{milling}(1+\frac{e^{\alpha }\;}{100})}{\left(Q_{milling}\times 60\right)}\;$$

where

*t_CNC_*_(*milling*)_ = the time taken for the milling operation (h);
*V_milling_* = the volume removed through the milling process (mm^3^);
*Q_milling_* = the material removal rate of the milling process (mm^3^/min);
*α* = the geometric complexity.

As CNC milling time exponentially increases with complex geometric features, the previously mentioned complexity factor was incorporated into the machining time calculations for milling processes. Furthermore, the cutting speed, feed rate, and depth of the cut were integrated into the material removal rate associated with each operation.

Similarly, the processing time required for BJP and DMLS is calculated as follows:

$$t_{BJP}=\frac{h_{component}\times \left(t_{layer}/3600\right)}{h_{layer}}+(t_{curing}/N)+(t_{sintering}/N)$$

where

*t_BJP_* = the time taken for the binder jetting process (h);
*t_curing_* = the curing time (h);
*t_sintering_* = the sintering time (h);
*h_component_* = the height of the component as per the build orientation (mm);
*h_layer_* = the height of the print layer (mm);
*t_layer_* = the time taken to print one layer (s);
*N* = the number of simultaneous builds.

$$t_{DMLS}=\frac{V\left(1+s\right)}{\left(\nu \times 60\right)}$$

where

*t_DMLS_* = the processing time for direct metal laser sintering (h);
*V* = component volume (mm^3^);
*S* = support material as a percentage of component volume;
*ν* = the build rate of the printer (mm^3^/min).

#### 4.1.2. Energy Consumption

Although it is imperative to consider environmental emissions at each individual stage of the manufacturing life cycle, the main focus of this research study was to evaluate the energy consumption resulting from the fabrication and material processing stages of the products. In scenarios where CNC machining and BJP were compared on a sustainability basis, the material types considered were stainless steel 304L, stainless steel 316, stainless steel 316L, stainless steel 420, and stainless steel 17-4. For the cases involving CNC machining and DMLS, stainless steel 316L and stainless steel 17-4 were considered.

The material preparation phases required for CNC machining are the initial mining of raw materials, conversion to primary metal, and the processing required to convert the initial stainless steel material to workpieces. In the case of BJP and DMLS, following the initial mining and conversion to the initial metal material, atomization is required to achieve the required powder metal. The average material embodied energy values (Btu/lb) for stainless steel billets and stainless steel powder were ascertained from the existing research [7,17]. The determination of total material processing energy for CNC machining is restricted to casting, rolling, and forging.

Since CNC machining results in a substantial amount of material waste, the impact of recycling metal chips on energy consumption was evaluated in this research study. Due to the fact that BJP and DMLS only utilize the amount of metal powder required by the component being built, material waste is minimal. Therefore, the impact of additive manufacturing waste material on energy consumption was assumed to be negligible.

The calculation procedures for determining the energy consumption of each manufacturing technology are depicted below.

▪ Material Processing Energy

$$E_{material\left(CNC\right)}=\left(M_{billet}-M_{waste}\times W\right)\times E_{embodied\left(billet\right)}+(E_{primary}\times M_{billet})+(E_{recycling}\times M_{waste}\times W)$$

where

*E_material_*_(*CNC*)_ = the material processing energy for CNC machining (kWh);
*E_embodied_*_(*billet*)_ = the material embodied energy for stainless steel (kWh/lb);
*M_billet_* = the mass of billet used for CNC machining (lb);
*E_primary_* = the embodied energy for workpiece fabrication (kWh/lb);
*E_recycling_* = the embodied energy for recycling the resultant waste (kWh/lb);
*W* = the percentage of waste recovered/recycled at the facility;
*M_waste_* = the mass of the resultant CNC machining waste (lb).

$$E_{material\left(DMLS\right)}=E_{material\left(BJP\right)}=M_{component}\times E_{embodied\left(powder\right)}$$

where

*E_material_*_(*DMLS*)_ = the material processing energy for DMLS (kWh/lb);
*E_material_*_(*BJP*)_ = the material processing energy for BJP (kWh/lb);
*M_component_* = the mass of the printed component (lb);
*E_embodied_*_(*powder*)_ = the material embodied energy for stainless steel (kWh/lb).

▪ Manufacturing Process Energy

To determine the amount of energy consumption related to the manufacturing process, it was imperative to use minimal empirical data. The energy consumption for DMLS was calculated using the generalized load factor [18], time for the build [12], and rated power of the machine.

$$E_{DMLS}=P_{DMLS}\times LF_{DMLS}\times (\frac{V\left(1+s\right)}{\left(\nu \times 60\right)}+t_{job\left(DMLS\right)})$$

where

*E_DMLS_* = the energy consumption of the DMLS process (kWh);
*P_DMLS_* = the rated power of the DMLS machine (kW);
*LF* = the load factor (%);
*V* = the component volume (mm^3^);
*S* = support material as a percentage of the component volume;
*ν* = the build rate of the printer (mm^3^/min);
*t_job_*_(*DMLS*)_ = the start-up time for the DMLS machine (h).

Miyanaji et al. [19] state that, at a constant saturation level, operating the infrared heater power of the BJP printer between 55% and 65% yields structures with required dimensional accuracies and appropriate characteristics sufficient for curing. For the purposes of this research, the heater power was considered to have a direct correlation with the input power of the BJP printer. For the energy consumption calculation of BJP printers, a 55% load factor was used as a generalized value.

$$\begin{array}{cc} E_{BJP}=(P_{BJP}\times & LF_{BJP}\times (t_{BJP}+t_{job\left(BJP\right)}))\\ & +(\frac{T_{average\left(curing\right)}}{T_{rated\left(curing\right)}}\times P_{rated\left(curing\right)}\times (t_{curing}/N))\\ & +(\frac{T_{average\left(sintering\right)}}{T_{rated\left(sintering\right)}}\times (t_{sintering}/N)) \end{array}$$

where

*E_BJP_* = the energy consumption of the BJP process (kWh);
*t_BJP_* = the time taken for the binder jetting process (h);
*t_job_*_(*BJP*)_ = the start-up time for the BJP machine (h);
*LF_BJP_* = the load factor for the binder jetting printer;
*P_BJP_* = the rated power of the binder jetting printer (kW);
*T_average_*_(*curing*)_ = the average temperature used in the curing process (°F);
*T_rated_*_(*curing*)_ = the maximum rated temperature of the curing oven (°F);
*P_rated_*_(*curing*)_ = the rated power of the curing oven (kW);
*t_curing_* = the curing time (h);
*T_average_*_(*sintering*)_ = the average temperature used in the sintering process (°F);
*T_rated_*_(*sintering*)_ = the maximum rated temperature of the sintering oven (°F);
*P_rated_*_(*sintering*)_ = the rated power of the sintering oven (kW);
*t_sintering_* = the sintering time (h);
*N* = the number of simultaneous builds.

According to the data obtained from numerous manufacturing facilities, the average load factor for metal CNC machining was generalized to be roughly 80%.

$$E_{CNC\left(milling\right)}=P_{CNC}\times LF_{CNC}\times (t_{CNC\left(milling\right)}+t_{job\left(CNC\right)})$$

where

*E_CNC_*_(*milling*)_ = the energy consumption of the CNC milling process (kWh);
*P_CNC_* = the rated power of the CNC machine (kW);
*LF_CNC_* = the load factor of the CNC machine (%);
*t_CNC_*_(*milling*)_ = the machining time for the CNC milling process (h).

The energy consumption for CNC turning and drilling can be calculated using the machining time for each process and substituting in the formula above.

For the analysis of energy consumption during the manufacturing processes, secondary finishing processes such as finish machining and painting were considered. The calculation procedure for secondary processing energy (*E_secondary_*) was adopted from [17].

$$\begin{array}{c} E_{DMLS\left(painting\right)}=E_{BJ\left(painting\right)}=E_{CNC\left(painting\right)}=(M_{initial})\times E_{\left(painting\right)}\;\\ E_{DMLS\left(finishmachining\right)}=E_{BJ\left(finishmachining\right)}=(M_{initial}-M_{final})\times E_{\left(finishmachining\right)}\\ \times E_{CNC\left(finishmachining\right)}=\left(0.1\times \frac{M_{billet}-M_{component}}{M_{billet}}\right)\times E_{\left(finishmachining\right)} \end{array}$$

* *Assumption: the finishing process for CNC machining removes 10% of the initial mass difference.*

where

*E_DMLS_*_(*painting*)_ = the energy consumption of painting for DMLS (kWh);
*E_BJP_*_(*painting*)_ = the energy consumption of painting for BJP (kWh);
*E_CNC_*_(*painting*)_ = the energy consumption of painting for CNC (kWh);
*M_initial_* = the mass of the initially printed component for DMLS/BJP (lb);
*M_final_* = the mass of the component after secondary processing DMLS/BJP (lb);
*M_billet_* = the mass of billet used in CNC machining (lb);
*M_component_* = the mass of the component for CNC machining (lb);
*E_(painting)_* = the embodied energy value for painting (kWh/lb);
*E_(finishmachining)_* = the embodied energy value for finish machining (kWh/lb);
*E_DMLS(finishmachining)_* = the energy consumption of finish machining for DMLS (kWh);
*E_BJP(finishmachining)_* = the energy consumption of finish machining for BJP (kWh);
*E_CNC(finishmachining)_* = the energy consumption of finish machining for CNC (kWh).

In addition to secondary processing, the energy consumption of post-processing (heat treatment) steps was considered in this research. It was assumed that all heat treatment devices were electric. The energy consumption of hot isostatic pressing and annealing can be calculated as follows:

$$E_{postprocessing}=P_{postprocessing}\times \frac{T_{zone\left(avg\right)}}{T_{rated}}\times t_{postprocessing}$$

where

*E_postprocessin_*_g_ = the energy consumption of the heat treatment process (kWh);
*P_postprocessing_* = the rated power of the heat treatment equipment (kW);
*T_zone_*_(*avg*)_ = the average zone temperature in the heat treatment equipment (°F);
*T_rated_* = the maximum rated temperature of the heat treatment equipment (°F);
*t_postprocessing_* = the time taken for the heat treatment process (h).

The total energy consumption for CNC machining, binder jetting, and direct metal laser sintering can be summarized as follows:

$$\begin{array}{c} E_{total\left(CNC\right)}=E_{CNC}+E_{secondary\left(CNC\right)}+E_{postprocessing\left(CNC\right)}\\ E_{total\left(BJ\right)}=E_{BJ}+E_{secondary\left(BJ\right)}+E_{postprocessing\left(BJ\right)}\\ E_{total\left(DMLS\right)}=E_{DMLS}+E_{secondary\left(DMLS\right)}+E_{postprocessing\left(DMLS\right)} \end{array}$$

#### 4.1.3. Economic Impact

In this research study, the economics pertaining to each manufacturing technique comprise equipment cost, material cost, labor cost, and cost due to energy consumption of the processes. For the purposes of this research study, it was considered important to include depreciation of equipment, installation and maintenance costs, and tax rates for equipment in determining the overall hourly cost rate for each machine. In order to account for the reduction in taxable income, the modified accelerated cost recovery system (MACRS) [20] was adopted in calculating the equipment cost. The overall costs associated with each manufacturing method were calculated as described below.

Operator involvement for a build entails concurrent activity time, independent activity time, and programming time required for the machine. The programming time for CNC and additive manufacturing was estimated as a function of the product’s geometric complexity [12]. Therefore, the total operator cost for CNC machining, BJP, and DMLS can be calculated as follows:

$$\begin{array}{c} L_{CNC}=\left(a+b+\left(20\times \alpha \right)\right)\times L_{cost}\\ L_{BJP}=\left(a+b+\left(10\times \alpha \right)\right)\times L_{cost}\\ L_{DMLS}=\left(a+b+\left(10\times \alpha \right)\right)\times L_{cost} \end{array}$$

where

*a* = the concurrent activity time;
*b* = the independent operator activity time;
*α* = the geometric complexity factor;
*L_cost_* = the hourly labor cost at the facility (USD/h);
*L_CNC_* = the total labor cost for CNC machining (USD);
*L_BJP_* = the total labor cost for BJP (USD);
*L_DMLS_* = the total labor cost for DMLS (USD).

Material costs are calculated as follows:

$$\begin{array}{c} M_{BJP}=M_{DMLS}=M_{component}\times M_{cost\left(powder\right)}\\ M_{CNC}=M_{billet}\times M_{cost\left(billet\right)} \end{array}$$

where

*M_BJP_* = the total material cost incurred during the BJP build (USD);
*M_DMLS_* = the total material cost incurred during the DMLS build (USD);
*M_CNC_* = the total material cost incurred during the CNC machining build (USD);
*M_component_* = the mass of BJP/DMLS component (lb);
*M_billet_* = the mass of billet used in the CNC machining process (lb);
*M_cost_*_(*powder*)_ = the cost of metal powder (USD/lb);
*M_cost_*_(*billet*)_ = the cost of metal billet (USD/lb).

Electricity costs for the manufacturing processes are quantified in the following manner:

$$\begin{array}{c} E_{cost\left(BJP\right)}=E_{total\left(BJP\right)}\times E_{cost}\\ E_{cost\left(DMLS\right)}=E_{total\left(DMLS\right)}\times E_{cost}\\ E_{cost\left(CNC\right)}=E_{total\left(CNC\right)}\times E_{cost} \end{array}$$

where

*E_cost_*_(*BJP*)_ = the electricity cost incurred for the BJP process (USD);
*E_cost_*_(*DMLS*)_ = the electricity cost incurred for the DMLS process (USD);
*E_cost_*_(*CNC*)_ = the electricity cost incurred for the CNC machining process (USD);
*E_total_*_(*BJP*)_ = the total energy consumption of BJP (kWh);
*E_total_*_(*DMLS*)_ = the total energy consumption of DMLS (kWh);
*E_total_*_(*CNC*)_ = the total energy consumption of CNC machining (kWh);
*E_cost_* = the blended electricity cost at the facility (USD/kWh).

In order to calculate the equipment depreciation costs, a 10-year class life was assumed for CNC machining, BJP, and DMLS equipment.

$$\begin{array}{cc} C_{cost}=(((C_{capital} & +C_{capital}\times C_{installation})+(C_{capital}\times C_{maintenance}{\left(1+C_{increase}\right)}^{10})\\ & -(C_{capital}(0.1\times R+0.18\times R+0.144\times R+0.1152\times R\\ & +0.0922\times R+0.0737\times R+0.0655\times R+0.0655\times R+0.0656\times R\\ & +0.0655\times R+0.0328\times R)))/10)/\left(8760\times UF\right) \end{array}$$

where

*C_cost_* = the hourly cost of the machine (USD/h);
*C_capital_* = the capital cost of equipment (USD);
*C_installation_* = the installation cost as a percentage of the capital cost;
*C_maintenance_* = the maintenance cost as a percentage of the capital cost;
*C_increase_* = the percentage annual increase in the maintenance cost;
*R* = the tax rate for manufacturing equipment;
*UF* = the utilization factor of equipment on an annual basis.

To calculate the machine cost incurred per build, the following equations were used:

$$\begin{array}{c} C_{CNC}=C_{cost\left(CNC\right)}\times t_{CNC}\\ C_{BJ}=C_{cost\left(BJ\right)}\times t_{BJ}\\ C_{DMLS}=C_{cost\left(DMLS\right)}\times t_{DMLS} \end{array}$$

where

*C_CNC_* = the total CNC machine cost for the build (USD);
*C_BJP_* = the total BJP machine cost for the build (USD);
*C_DMLS_* = the total DMLS machine cost for the build (USD);
*C_cost_*_(*CNC*)_ = the cost per hour for the CNC machine (USD/h);
*C_cost_*_(*BJP*)_ = the cost per hour for the BJP machine (USD/h);
*C_cost_*_(*DMLS*)_ = the cost per hour for the DMLS machine (USD/h);
*t_CNC_* = the processing time for CNC machining (h);
*t_BJP_* = the processing time for BJP (h);
*t_DMLS_* = the processing time for DMLS (h).

The total economic impact of the manufacturing processes (CNC machining, BJP, and DMLS) was estimated by combining material, labor, electricity, and machine costs relevant to the fabricated component.

#### 4.1.4. Impact of Auxiliary Material on Sustainability

Intuitively, CNC machining consumes a larger proportion of auxiliary material due to the nature of its process. The auxiliary systems with the highest sustainability impact in CNC machining have been considered to be cutting tools and cutting fluids. In this research, the considered cutting tool types for machining stainless steel were carbide and ceramic, due to their significant usage in industry. Ceramic cutting tools have a much higher resource efficiency than carbide tools during the high-speed machining of hard materials in dry conditions [21].

According to [22], dry machining is recommended for milling operations with ceramic materials. Due to the absence of cutting fluid, dry machining would result in a reduced burden on the environment and less adverse impact on worker safety due to decreased exposure to harmful chemicals. However, turning processes would require the use of cutting fluid. In cases where dry machining is not viable, minimum lubrication is utilized by the use of ceramic tools. However, ceramic tools require precious resources and higher energy expenditure during production and recycling due to added processing steps such as sintering and other heat treatment methodologies [21].

In the case of carbide tools, upon reaching tool life, roughly 40 percent of the recycled material is used for the production of new cutting tools, and the remainder is used for mining tools. The use of chemical recycling processes allows for the reuse of almost 100% of disposed carbide-cutting tools for the production of new tools. Therefore, carbide tools undergo either open- or closed-loop recycling during their end-of-life phase. It has been proven that cutting tools manufactured using recycled tool material results in 75% less energy consumption than that of virgin material-based production [23].

During the binder jetting process, the auxiliary material used consists of binder fluid and cleaning fluid. In this research, only the sustainability impact of binder fluid was considered. Since all binder material is consumed during the printing process, there is no requirement for recycling or disposal. The auxiliary material consumption of DMLS is only due to inert gas utilization and compressed air. Since compressed air is used at low flow rates, its impact on sustainability was neglected. It is important to note that the sustainability impact due to the flow rates of the auxiliary materials used in BJP and DMLS was not taken into consideration as they are highly variable among machines and processes.

### 4.2. Application of Analytical Hierarchy Process to Knowledge Base

The knowledge base was used in conjunction with a scale of relative importance [10] to designate ratings for each technology as per their auxiliary material consumption. The eigenvector (*p*) depicts the relative weights between each criterion calculated by taking the arithmetic mean of all criteria. The following priority weights for the sustainability criteria were obtained by normalizing the principal eigenvectors of the matrix, as described in Section 3.4. According to the methodology utilized in this research, the calculation of judgment matrix weights for sustainability indicators is only required in the initial phase of the algorithm. Upon the calculation of these weights/priority vectors, as represented in Table 2, the values were directly incorporated into the logic block rules of the expert system.

The next step of the AHP is the evaluation of manufacturing processes (BJP/CNC machining and DMLS/CNC machining) considering auxiliary material usage. As evident from the information in Section 4.1.4, CNC machining has a higher sustainability impact due to the significant use of auxiliary materials in this process compared with BJP and DMLS, and DMLS is the most sustainable technology. Therefore, the scores were assigned in a manner that reflects better performance. Weights of the judgment-based matrices are predominantly based on the type of cutting fluid and cutting tool utilized in the CNC process. Additionally, regrinding/reusing cutting tools was considered to have a favorable impact on sustainability. For simplicity, a range of manufacturing scenarios in which process parameters may vary in terms of auxiliary systems were considered for the allocation of weights. A comparison matrix is shown in Table 3, as an example of DMLS and CNC machining for the scenario in which ceramic tools were used with minimal coolant fluid or dry machining.

Tools were reground upon breaking or reaching tool life. The auxiliary material weights for each manufacturing technology were computed based on the methodology described in Section 3.4. However, for the purposes of this research, the ratings were modified to be represented on a scale ranging from 1 to 5.

### 4.3. Design of Expert Systems

#### 4.3.1. MSUSTAIN1 Expert System

The system consists of 253 nodes and includes static lists, numeric values, and confidences. User input data comprise information that is specific to the manufacturing facilities or would originate from the associated CAD models. As per the design of the system, the user can opt to evaluate either binder jetting and CNC machining, or direct metal laser sintering and CNC machining for the specific component being manufactured. The system first determines if the user-defined component satisfies the product parameters equally viable for the two manufacturing technologies under consideration. If the product parameters are satisfied, the system then proceeds to evaluate the capability of each technology in achieving the system-level requirements based on utilized process parameters. For the CNC machining time, the user is given the option to enter specific cutting speeds, feeds, and depths for each operation, or input the time as simulated using CAD software.

**Figure 4:**
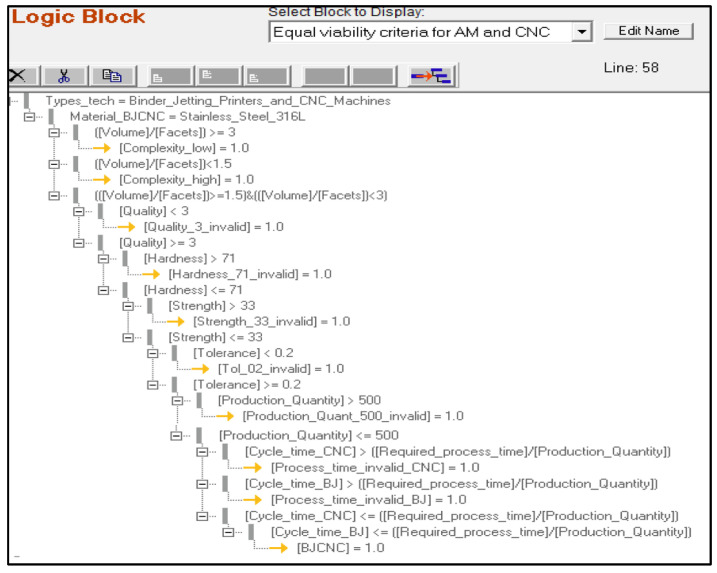
Design of main logic block rules for SS316L, BJP vs. CNC.

**Figure 5:**
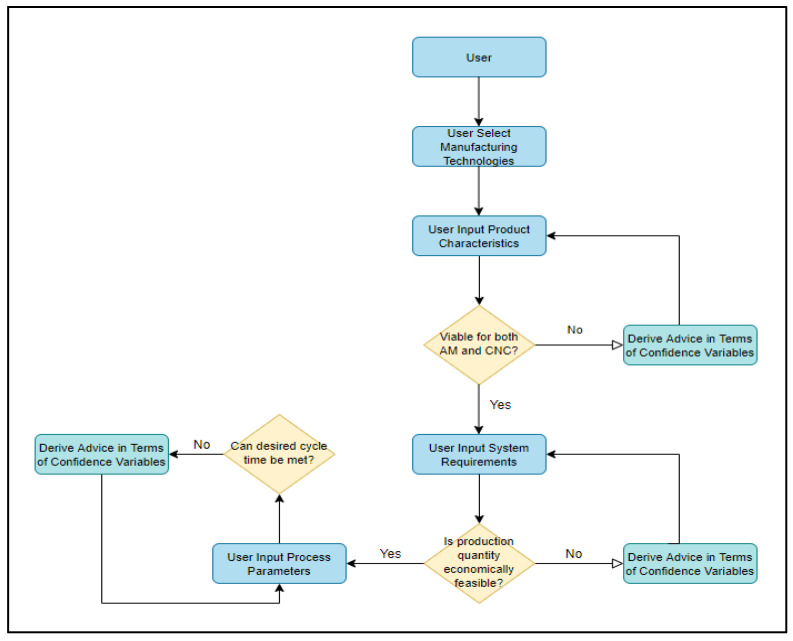
Algorithm for MSUSTAIN1.

If all product, process, and system criteria for both technologies are met, the system informs the user and advises them to proceed to MSUSTAIN2. If a certain parameter is outside of the scope of these regions, the user is informed and requested to alter the input accordingly. Figure 4 depicts the standard logic tree structure utilized in MSUSTAIN1 for deriving various confidence variables corresponding to user-defined product, process, and system parameters. The algorithm for the MSUSTAIN1 is represented in Figure 5.

#### 4.3.2. MSUSTAIN2 Expert System

The system is built using 314 nodes and consists of crisp information (100% confidence levels). The necessary equations and algorithms are incorporated into the logic blocks such that the user is informed of the cost (USD), energy consumption (kWh), and sustainability rating (on a scale of 1 to 5) for auxiliary material. The expert system logic is separated into 11 blocks that carry out specific functions such as the calculation of energy consumption for the primary process, finishing, and post-processing; cost evaluation; and the determination of auxiliary material weights using AHP. Contrary to MSUSTAIN1, MSUSTAIN2 does not require the determination of confidence variables. Instead, the command block is designed to derive values for energy (kWh), cost (USD), and auxiliary material weights using AHP.

**Figure 6:**
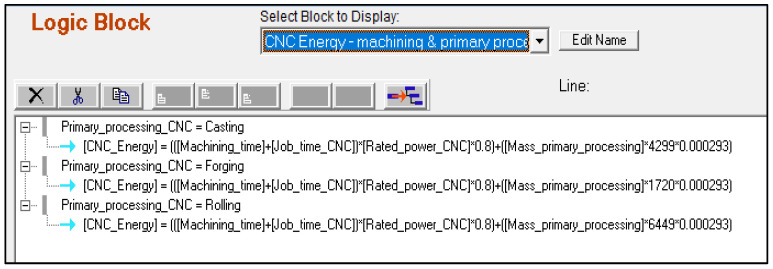
Logic block sample for the energy consumption of the CNC process.

**Figure 7:**
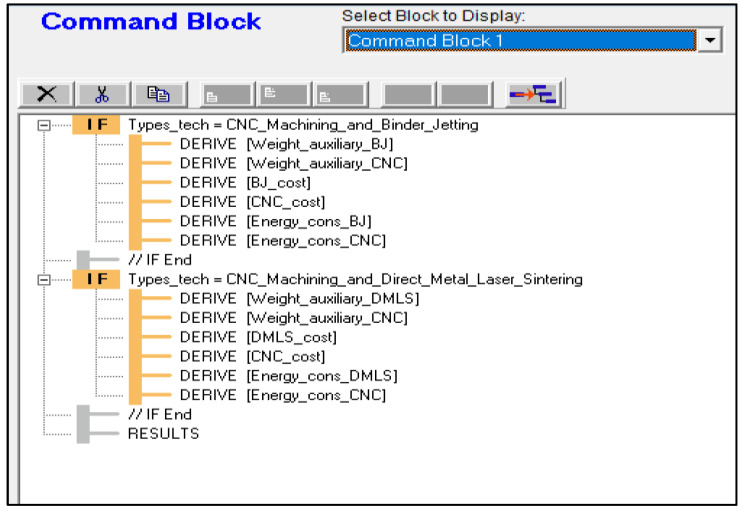
Command block structure for MSUSTAIN2.

To be consistent with MSUSTAIN1, the user has to initially choose between BJP and CNC machining, or DMLS and CNC machining to compare their sustainability. The system then inquires the user regarding product and process parameters required by each manufacturing technique. Figure 6 presents the logic block sample for the energy consumption of the CNC process. Figure 7 depicts the structure of the Corvid^®^ Command Block Window for MSUSTAIN2.

**Figure 8:**
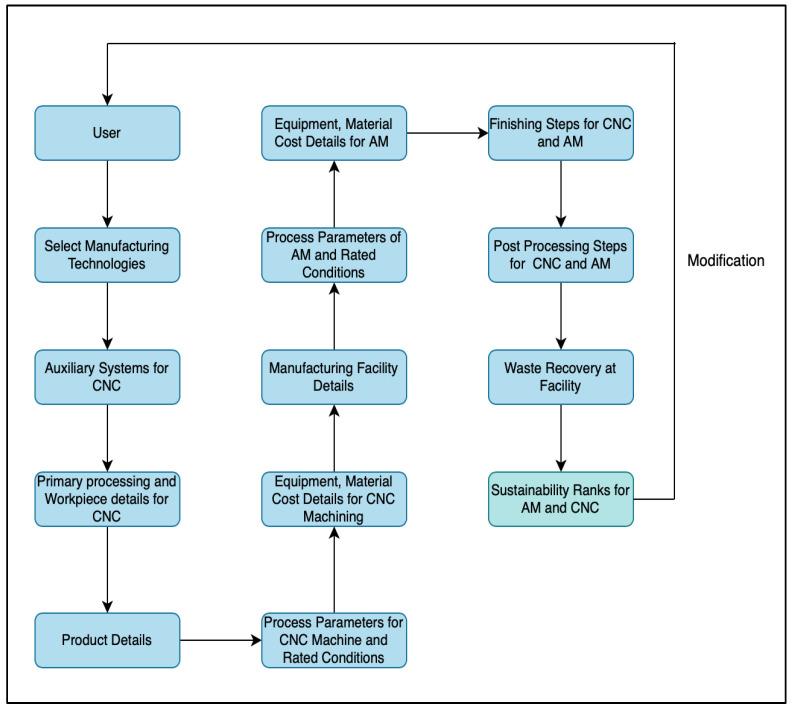
Algorithm for MSUSTAIN2.

Conditional inferencing is utilized to derive sustainability performance values based on the type of technologies being compared. The goal of the system is defined such that variables corresponding to the values of sustainability indicators are derived using backward chaining. The algorithm for MSUSTAIN2 is represented in Figure 8.

## 5. Verification and Analysis of Model

The robustness of the knowledge base and logical rules built into the expert systems were tested by further analysis and verification. To test the accuracy of the expert systems, it was considered imperative to fabricate a test part using CNC machining and binder jetting and compare the output results of the programs to the actual data retrieved during manufacturing.

The details of the case study are discussed in the following sections. The component selected was a reduced-scale model representing the control arm of a vehicle suspension system. Stainless steel (SS) 316L was selected due to its high strength and corrosion resistance. The material properties, dimensions, and tolerances for the control are depicted in Table 4. Secondary/tertiary processing steps such as finishing and heat treatment were not considered in the case study due to limitations in equipment.

### 5.1. Energy and Material Consumption of the BJP Process

The real-time electrical data recording during each stage of fabrication by ExOne Innovent BJP printer was extracted using a series of instruments such as current transducers, data loggers, and a hand-held multimeter. The total energy consumption of the BJP process during the production of a single part (iteration 1) of the control arm consists of energy consumption in the printing, curing, and sintering processes.

During the second iteration (six parts) of the experiment, the energy consumption of the curing and sintering processes remained identical to that of the former. The summary of energy consumption during each iteration of the process is shown in Table 5. Data collected from the manufacturing processes indicate that the energy intensity per part can be significantly reduced via the build volume optimization of the printer. As the material waste is negligible in the BJP process, the material used during fabrication can be estimated as being equal to the volume of the component. In this case, it was 7600 mm^3^.

### 5.2. Energy and Material Consumption of CNC Process

The stainless steel part was manufactured using a Tormach 1100M four-axis CNC mill. The energy consumption during the production of a single unit of the control arm was found to be 3.9 kWh. CNC machining processes result in a large amount of waste material due to the generation of chips. The dimensions of the workpiece were selected such that minimal waste material would be generated during production. Material allowances required by the process were also considered. The volume of the workpiece was 147,484 mm^3^. The volume of waste material was the difference between the volume of the workpiece (147,484 mm^3^) and the volume of the component (7600 mm^3^), which was 139,884 mm^3^. This was equal to the mass of 1123.3 g (density of SS316 = 8.03 × 10^−3^ g/mm^3^).

### 5.3. Comparison of Energy, Waste Material, and Carbon Emissions

Based on the results obtained from the pilot study, a comparison of the energy, waste material, and carbon emissions of the manufacturing process was conducted.

In order to determine the associated carbon emissions, the average annual CO_2_ emissions factor for electricity generated (0.9904 lbsCO_2_/kWh) [24] in the United States was considered. A summary of the results is tabulated in Table 6.

### 5.4. Analysis and Verification of Case Study

The expert systems were used to evaluate the fabrication of the control arm using binder jetting and CNC machining. The product and process parameters for both expert systems reflect the information provided in the previous sections pertaining to the fabrication of the control arm.

However, due to the requirement of cycle time calculation in MSUSTAIN1, the concurrent activity time, the independent operator activity time, the number of machines assigned per operator, the required processing time, and the required production quantity were assumed based on a hypothetical manufacturing scenario. The input product and system parameters for viability ES are shown in Table 7 and Table 8, respectively.

**Figure 9:**
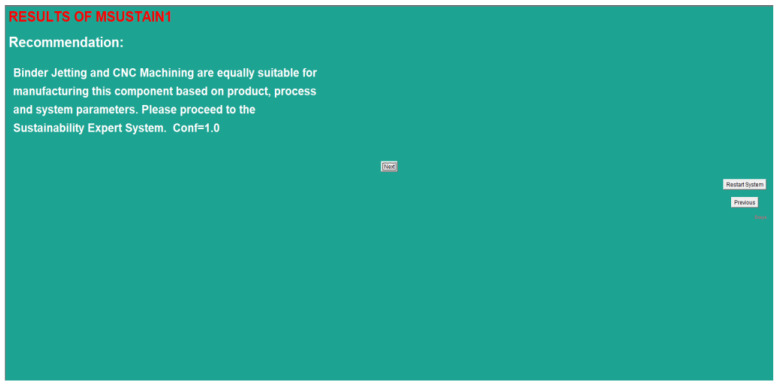
Results screen of MSUSTAIN1 for a case study component.

By evaluating the input data based on the built-in logical rules, MSUSTAIN1 was capable of yielding the results shown in Figure 9.

It is clear that the product parameters listed in Table 7 are within the equal viability criteria for BJP and CNC as per Section 4.1.1. Upon evaluating the logical rules associated with the product parameters, MSUSTAIN1 then proceeded to calculate the attainable cycle times for BJP and CNC based on process parameters. The cycle times for BJP and CNC based on the process parameters were 5.06 h and 3.65 h, respectively. The cycle time required by the customer in this hypothetical scenario was 6.72 h (Table 8). Therefore, the results shown in Figure 9 are justified.

**Figure 10:**
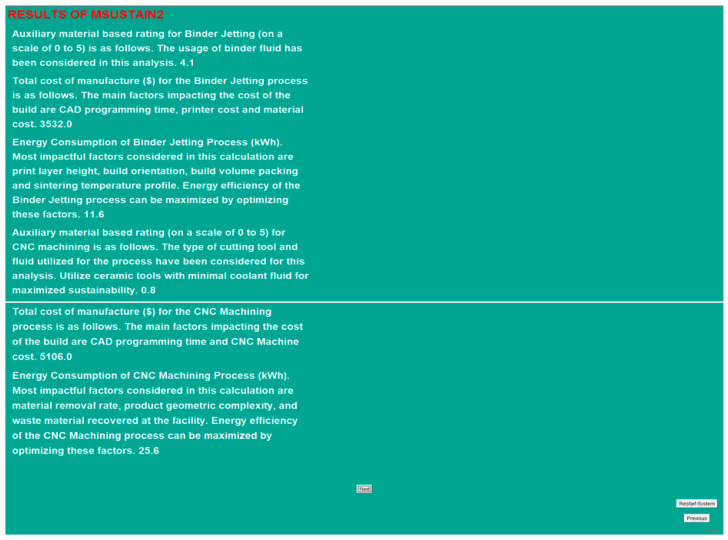
Results screen of MSUSTAIN2 for a case study component.

In order to assign the values depicted in Figure 10, the MSUSTAIN2 system was used to carry out the calculation procedures described earlier. The energy consumption values for BJP and CNC machining were determined to be 11.6 kWh and 25.59 kWh, resulting in 55% energy savings from the utilization of BJP for the manufacturing process. The costs involved in fabricating the component were found to be USD 3,532 for BJP and USD 5,106 for CNC machining, resulting in 31% cost savings due to the use of BJP for the process. Although material and equipment costs for CNC machining are much lower than BJP, the cost-effectiveness of using BJP for this specific process can be explained due to its minimal labor involvement and reduced energy consumption.

**Figure 11:**
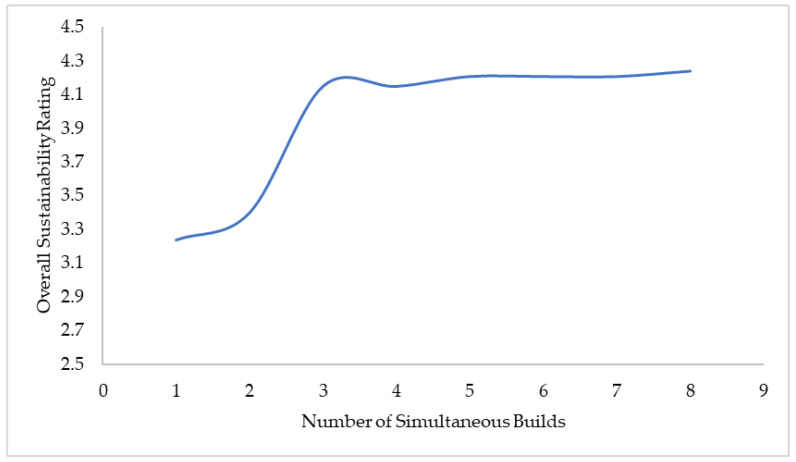
Variation in overall sustainability rating for BJP due to build volume utilization.

**Figure 12:**
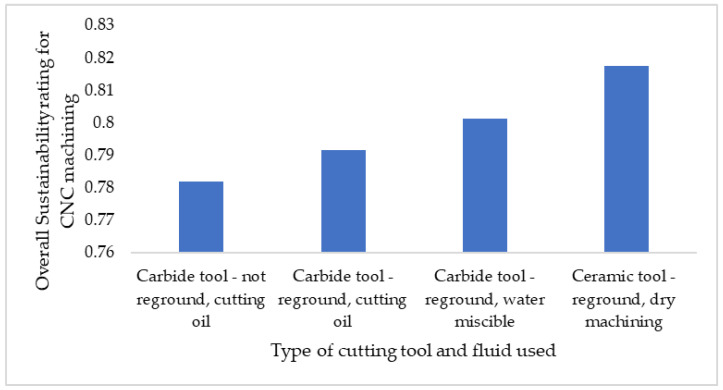
Variation in overall sustainability rating for CNC due to auxiliary material types utilized.

Sensitivity analysis was conducted for the parameters deemed most crucial to the sustainability performance of each manufacturing technology, and it was limited to the primary and secondary stages of the manufacturing processes. It is clear that the sustainability performance of BJP in the domain of auxiliary material is superior to that of CNC machining. The variation in the overall sustainability rating for BJP due to build volume utilization is presented in Figure 11. However, by using ceramic tooling with minimal coolant/dry conditions and adopting best practices such as tool regrinding, the sustainability rating for the CNC machining of the control arm can be improved, as depicted in Figure 12.

## 6. Conclusions and Future Work

The developed expert systems were verified by analyzing the fabrication process of a stainless steel 316L component. For this purpose, the outputs of the expert systems were compared against the information found in the existing literature and data collected during the fabrication of an automotive control arm using binder jetting and CNC machining. According to the results of the case study, the energy consumption algorithms of MSUSTAIN2 yielded accuracy levels of 96.7% and 90.7% for BJP and CNC machining, respectively. BJP was found to have more favorable sustainability indicators for manufacturing a metal component feasible for each technology. From authors field experience, this research can aid the efforts required considerably within manufacturing facilities implementing energy management and environmental standards such as ISO 50001/Superior Energy Performance/ISO14001.

Although the knowledge bases contained in the expert systems are robust enough for the purposes of this research, further improvements can be made. Considerations for future work are stated below.

➢ Incorporate the impact of additive manufacturing and CNC machining on supply chains, the product use phase/end-of-life energy consumption, worker safety, and health;
➢ Expand the analysis criteria to other metal additive manufacturing techniques;
➢ Include additional CNC machining operations;
➢ Implement an algorithm that considers intricate details of geometric features for the estimation of product geometric complexity;

## Figures and Tables

**Table 1:** Geometric complexity factors for CNC machining.

Ratio	Complexity Factor
Volume–Facets ≥ 3	2.5
1.5 ≤ Volume–Facets < 3	5
0.5 ≤ Volume–Facets < 1.5	7.5
Volume–Facets < 0.5	10

**Table 2:** The judgment matrix for criteria.

Criteria	Cost	Energy	Auxiliary Material	Priority Vector
Cost	1	5	7	0.6758
Energy	1/5	1	7	0.2595
Auxiliary Material	1/7	1/7	1	0.0647
Sum	1.34	6.14	15	1.00

**Table 3:** Comparison matrix for DMLS and CNC in terms of auxiliary material.

Auxiliary Material	Direct Metal Laser Sintering	CNC Machining	Priority Vector
Direct Metal Laser Sintering	1	4	0.8
CNC Machining	1/4	1	0.2
Sum	1.25	5	1.00

**Table 4:** Control arm material properties, dimensions, and tolerances.

Material	Stainless Steel 316L powder–BJP Stainless Steel 316L annealed bar–CNC
Mass	61 g
Volume	7600 mm^3^
Features and Dimensions	Chamfers: 3.50 mm × 3.50 mm
	Fillets: Ø 1.5 mm
	Counterbore: Ø 3 ± 0.2 mm, Ø 5.20 ± 0.2mm
	Rectangular slot: 12.50 ± 0.2 mm × 24.70 ± 0.2 mm
	Small holes: 2.00 ± 0.2 mm, 3.00 ± 0.2 mm
	Notch: 26.00 ± 0.2 mm

**Table 5:** Energy consumption of BJP process.

Stage of BJP Process	Energy Consumption (kWh)	
	Iteration 1 (1 Part)	Iteration 2 (6 Parts)
Printing	0.5	2.5
Curing	6	6
Sintering	46.4	46.4
Total	52.9	54.9
Energy Intensity/Part	52.9	9.15

**Table 6:** Summary of energy, material consumption, and carbon emissions.

Manufacturing Process	Production Units	Energy Consumption (kWh)	Carbon Emissions (lbsCO_2_)	Waste Material (lb)
Binder Jetting	1	9.15	9.06	0
CNC Machining	1	3.9	3.86	2.48

**Table 7:** Input product parameters for viability ES.

Input Parameter	Value
Material Type	Stainless Steel 316L
Volume of component (mm^3^)	7600
Number of facets in CAD model	4380
Surface quality (μm)	5
Hardness (HRB)	60
Strength (ksi)	30
Tolerance/dimensional accuracy (mm)	±0.2

**Table 8:** Input system parameters for viability ES.

Input Parameter	Value
Production quantity	50
Required process time (h)	336

## Data Availability

The data are not publicly available due to non-disclosure agreements.
